# Dietary Strategies to Modulate the Health Condition and Immune Responses in Gilthead Seabream (*Sparus aurata*) Juveniles Following Intestinal Inflammation

**DOI:** 10.3390/ani12213019

**Published:** 2022-11-03

**Authors:** Carla Teixeira, Diogo Peixoto, Mariana Hinzmann, Paulo Santos, Inês Ferreira, Gabriella V. Pereira, Jorge Dias, Benjamín Costas

**Affiliations:** 1 CIIMAR—Interdisciplinary Centre of Marine and Environmental Research, University of Porto, Novo Edifício do Terminal de Cruzeiros de Leixões, Avenida General Norton de Matos, s/n, 4450-208 Matosinhos, Portugal; 2 SPAROS Lda.—Área Empresarial de Marim, Lote C, 8700-221 Olhão, Portugal; 3 ICBAS—Abel Salazar Biomedical Sciences Institute, University of Porto, Rua Jorge de Viterbo Ferreira, 228, 4050-313 Porto, Portugal; 4 i3S—Institute for Research and Innovation in Health, Rua Alfredo Allen, 208, 4200-135 Porto, Portugal

**Keywords:** dextran sodium sulphate, intestinal inflammation, β-glucans, curcumin

## Abstract

**Simple Summary:**

Feed additives are known to have biological proprieties that can improve fish health. This work assessed the effect of two feed additives (*Phaeodactylum tricornutum* extracts rich in β-glucans and curcumin) on the gilthead seabream health condition, and its modulatory effects following dextran sodium sulphate (DSS) administration as a chemical inducer of intestinal inflammation. While minor immune-enhancing changes were observed among fish fed dietary treatments at the end of the feeding trial, after the inflammatory stimulus, the feed additives were able to alleviate, to some extent, the DSS-induced effects at both the intestinal and systemic levels.

**Abstract:**

Several feed additives have proved to be beneficial in eliciting fish health. Β-glucans and curcumin are compounds with immunomodulatory capacities known to increase growth performance, stimulate immunity, improve general health, and enhance disease resistance in fish. The present study aimed to evaluate the effects of dietary *Phaeodactylum tricornutum* extracts rich in β-glucans and curcumin on gilthead seabream health status prior to and following an intestinal inflammatory stimulus. Three experimental diets were formulated: a practical commercial-type diet (CTRL), a CTRL diet supplemented with 1% microalgae-derived β-glucans extract (BG), and a CTRL diet supplemented with 0.2% of curcumin (CUR). After 30 days of the feeding trial, fish were sampled and subjected to an oral administration of 1% dextran sodium sulphate (DSS) to induce intestinal inflammation. Four groups were considered: a group of fish continued to be fed on the CTRL diet while the remaining groups were exposed to DSS, including CTRL-D (CTRL + DSS), BG-D (BG + DSS), and CUR-D (CUR + DSS), for 6 days. Growth, plasma and gut humoral immunity, liver and gut oxidative stress biomarkers, and intestinal gene expression were evaluated. No significant differences were found in growth after 30 days of feeding; however, seabream fed BG had decreased anti-protease activity and nitric oxide concentration in plasma while those fed CUR had increased mRNA levels of the *tnfα*, *csf1r*, and *hep* genes compared to those fed CTRL. After the inflammatory stimulus, hematocrit was enhanced in fish fed BG-D and CUR-D while red blood cell counts increased in those fed CTRL-D. Superoxide dismutase activity decreased in the intestine of all DSS groups while lipid peroxidation increased in the gut of fish fed CTRL-D and BG-D compared to CTRL. Moreover, the mRNA expression levels of *csfr1* and *sod* decreased in fish fed CTRL-D and BG-D compared to CTRL, respectively. Despite the mild intestinal inflammatory condition induced by DSS, CUR was able to partially ameliorate its effects, improving the hematological profile and assisting against the oxidative stress.

## 1. Introduction

Gilthead seabream (*Sparus aurata*) production has scaled up in the last years due to its high demand and commercial value, translating into high stocking densities and stressful rearing conditions, which contributes to reduced growth, immunosuppression, higher fish mortality, and huge economic losses [1,2,3]. Therefore, improving the aquaculture practices, fish health, and disease resistance of farmed fish is crucial [4]. The demand for high-productivity aquaculture is placing pressure on the available supplies for aquafeed formulations, and alternative ingredients are necessary to maintain the sustainability of this industry [5,6]. Unconventional alternatives to fish meal and fish oil are being extensively tested in aquaculture [6]. However, a lack of palatability, amino acid imbalance, poor digestibility, high amounts of fiber and non-starch polysaccharides, and anti-nutritional factors (saponins, alkaloids, tannins) can narrow their use in aquafeed formulations [7]. In addition, anti-nutritional factors can induce intestinal damage in fish and cause intestinal inflammation (enteritis), which reduces the intestinal absorptive capacity, increases mucus secretion, induces hyperpermeability, enhances leucocyte infiltration, and activates the pro-inflammatory cytokines (TNFα, IL1β, IL8) [8,9,10].

Dextran sodium sulphate (DSS) is a water-soluble sulphated polysaccharide detergent with anti-coagulant proprieties that creates a dysfunction in the intestinal barrier, with the production of toxic local effects, misbalance of the intestinal flora (dysbiosis), dissemination of the pro-inflammatory contents, and dysregulation of the macrophage function [11,12]. Ingestion of DSS is a common method used to induce intestinal inflammation in several animal models [11,12,13,14,15,16,17,18], including in fish [19,20,21,22,23,24,25,26]. DSS penetrates the mucosal membrane in the intestine and invades local macrophages, but it can reach other organs and tissues [27].

A common practice to achieve economic sustainability and maximum production in aquaculture involves the use of functional feeds [4]. A functional feed is a well-balanced diet supplemented with feed additives [4]. Several feed additives (probiotics, prebiotics, nucleotides, phytochemicals) are known to be beneficial in improving fish health [28,29,30,31,32]. Functional feeds can improve innate immunity and disease resistance and are regarded as prophylactic alternatives to antibiotics and chemotherapeutics [4,30,33].

β-glucans are polysaccharides of glucose molecules, linked by either a β-(1,3) or β-(1,4) liner backbone, with none, short, or long β-(1,6) sidechain branches, that usually originate from the cell wall of plants, fungi, bacteria, or seaweeds, and have different structures, sizes, solubility, activities, and immunomodulatory effects [2,4,34]. β-glucans can be used as immunostimulants, adjuvants, or prebiotics and be administrated orally, intraperitoneal, or by immersion [1,4,34]. Diatoms (*Bacillariophyta*) are photosynthetic eukaryotes found in aquatic environments [35]. In diatoms, the carbohydrate content is mainly in the form of polysaccharides and divided into three groups: storage β-glucans (chrysolaminarin, water-soluble), cell wall polysaccharides (glucuronommannans, insoluble in water), and extracellular polysaccharides (chitin, water-soluble) [35]. In *Phaeodactylum tricornutum*, the main β-glucan reserve polysaccharide is chrysolaminarin (β-1,3-glucan with β-1,6-branching) [35], similar to laminarin though with lacking a terminal mannitol residue [36]. Chrysolaminarin is a low-molecular-weight soluble product of the photosynthesis process that is stored in the cell vacuoles [35,36,37]. Chrysolaminarin-enriched extracts present strong antioxidant activity and immunomodulatory effects in fish [37], including in the gilthead seabream [38], and have been associated with higher survival rates and higher average weight in fish larvae [39].

Curcumin is a phytochemical compound obtained from the rhizomes of the turmeric herb (*Curcuma longa*) known to have biological properties, such as antioxidant, anti-inflammatory, antimicrobial, anticancer, and immunostimulant properties, and is also involved in growth promotion and increased disease resistance in fish [40,41,42,43,44,45,46,47,48,49,50,51]. After ingestion, curcumin is transformed in metabolites and about 75% of orally administered curcumin is excreted in feces [40]. The turmeric herb is rich in phenolic compounds (curcuminoids) known to be strong antioxidants [40].

DSS can be used in fish as an inflammatory model to study intestinal inflammation. The present study aimed to produce a controlled inflammatory process to evaluate the effects of two feed additives (*Phaeodactylum* β-glucans and curcumin) on gilthead seabream’s health condition following an intestinal inflammatory stimulus. Several biomarkers related to growth, immunity, oxidative stress, and gene expression were assessed to evaluate the inflammatory effects produced by DSS and the potential modulatory proprieties of the feed additives on intestinal and health-related parameters.

## 2. Materials and Methods

### 2.1. Experimental Diets

Three diets were formulated to be isonitrogenous (46% crude protein) and isolipidic (18% crude fat) (Table 1). A practical commercial-type diet was used as the control (CTRL) whereas two experimental diets based on CTRL were supplemented with either a 0.2% of curcumin extract (CUR) or 1% of an extract of *P. tricornutum* rich in β-glucans (BGs). Chrysolaminarin-rich biomass from *P. tricornutum* (SAG 1090 1b) was grown under nitrogen-depleted conditions in flat panel airlift reactors and was harvested and concentrated via centrifugation to 250–270 g L^−1^ (Clara 20, Alfa Laval, Lund, Sweden) and frozen at −20 °C. Afterwards, the biomass was thawed and diluted to 100 g L^−1^ with deionized water, and cells were disrupted with a ball mill (PML-2, Bühler, Uzwil, Switzerland). The disrupted biomass was then centrifuged, and the β-glucan-rich supernatant was freeze-dried (Avanti J-26 XP, Beckman Coulter, Brea, CA, USA). The β-glucans content in this algae extract was 37%.

Diets were manufactured by extrusion at SPAROS Lda. (Olhão, Portugal). All powder ingredients were mixed according to the target formulation in a double-helix mixer (model 500L, TGC Extrusion, Roullet-Saint-Estèphe, France) and ground (below 400 µm) in a micropulverizer hammer mill (model SH1, Hosokawa-Alpine, Augsburg, Germany). Diets (pellet size: 3.0 mm) were manufactured with a twin-screw extruder (model BC45, CLEXTRAL, Firminy, France) with a screw diameter of 55.5 mm. Extruded pellets were dried in a vibrating fluid bed dryer (model DR100, TGC Extrusion, Roullet Saint Estephe, France). After cooling, oils were added by vacuum coating (model PG-10VCLAB, Dinnissen, The Netherlands). A second set of all diets was also made with the incorporation of 1% DSS (MW: 40 KDa; TdB Labs AB, Uppsala, Sweden) by top-coating. Diets were stored in a temperature-controlled room.

### 2.2. Fish and Experimental Design

The experimental trial was executed at the CIIMAR (Interdisciplinary Centre of Marine and Environmental Research) facilities (Matosinhos, Portugal). Gilthead seabream juveniles obtained from Sonríonansa, S.L. (Pesués Cantabria, Spain), weighing 34.0 ± 0.5 g, were randomly distributed in 12 tanks with a 250 L water capacity (25 fish each tank) and maintained in a seawater recirculating system. A photoperiod of 12 h light 12 h dark^−1^ was applied. Water dissolved oxygen was around 6.3 ± 0.5 mg L^−1^, the water temperature was around 20.6 ± 0.5 °C, pH was around 7.7 ± 0.2, and salinity was around 35.3 ± 0.5 g L^−1^. All animals were acclimatized to the experimental conditions for 10 days and fed with the CTRL diet twice a day, ad libitum.

**Figure 1:**
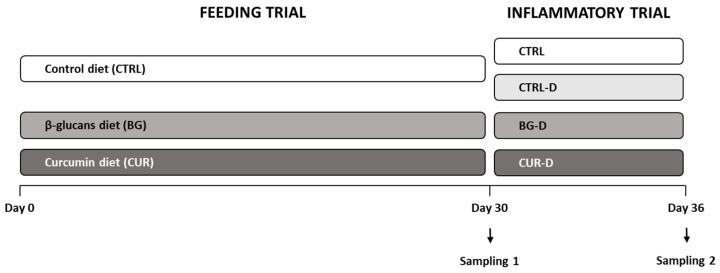
Experimental design of the feeding and gut inflammation trials. CTRL (control diet), BG (β-glucans diet), CUR (curcumin diet), CTRL-D (control diet + dextran sodium sulphate—DSS), BG-D (β-glucans diet + DSS), CUR-D (curcumin diet + DSS).

After the acclimatization period, a feeding trial was performed, and three dietary treatments were randomly assigned. Fish from 6 tanks were fed the CTRL diet, whereas fish from the other 6 tanks were fed the BG or CUR diet in triplicates. Seabream juveniles were fed twice a day, ad libitum, and the feeding trial lasted for 30 days (Figure 1).

After the feeding trial, dextran sodium sulphate (DSS)-induced inflammation in the intestine was performed using 1% of DSS in the diet. For this purpose, four dietary treatments were used in triplicates. Three tanks from the CTRL group remained with the CTRL diet, whereas the other 3 tanks shifted to the CTRL diet supplemented with DSS (CTRL-D). The BG and CUR groups were also fed the BG and CUR diets supplemented with DSS (BG-D, CUR-D). The fish were fed twice a day, at a ration of 1.5% body weight day^−1^, and the inflammatory trial lasted for 6 days (Figure 1).

All fish procedures followed Portuguese and European guidelines on the protection of animals used for scientific purposes, such as Directive 2010/63/UE and Decreto-Lei n.° 113/2013 de 7 de Agosto, and FELASA category B and C recommendations.

### 2.3. Sample Collection

At the end of the feeding and inflammatory trials (day 30 and 36, respectively), samples were taken to evaluate the growth, health status, and inflammatory condition. Four fish per tank (*n* = 12) were randomly selected, anesthetized by immersion in 2-phenoxyethanol (1500 ppm; Sigma, St. Louis, MO, USA), and sampled for weight measurements, blood, and tissue. Blood was collected from the caudal vein using heparinized syringes (2500 U.I., Braun; 25 G, 1 mL). Then, the fish were opened, and the internal organs were placed in a petri dish. Liver samples were collected and stored at −80 °C to evaluate oxidative stress. Sections of 0.5 cm of the anterior intestine were collected, flushed with sodium phosphate buffer, and stored at −80 °C to evaluate the immunity and oxidative stress or stored in RNAlater^TM^ (Sigma) at −20 °C for gene expression. 

### 2.4. Hematological Profile

The hematological profile was verified, and the hematocrit percentage (HT), total red blood cell (RBC) counts, and total and differential white blood cell (WBC) counts were performed according to Machado et al. [52]. The hemoglobin (HG) content (Hemoglobin—Drabkin—colorimetric, Spinreact) was quantified. The mean corpuscular volume (MCV), the mean corpuscular hemoglobin (MCH), and the mean corpuscular hemoglobin concentration (MCHC) were calculated as described by Machado et al. [52]. From the homogenized blood, blood smears were prepared following Machado et al.’s [52] protocol, and the identification of neutrophiles, through the presence of peroxidase activity, was carried out according to Afonso et al. [53]. Additionally, the blood smears were stained with Wright’s stain (Haemacolor, Merck) to perform differential WBC counts, as described by Machado et al. [52]. 

### 2.5. Plasma and Intestinal Humoral Immune Parameters

Immune-related parameters were analyzed in the plasma and intestine. The intestinal samples were homogenized (1:10) with K phosphate buffer (KPB) (K_2_HPO_4_ 0.1 M, KH_2_PO_4_ 0.1 M, pH 7.4, Sigma) using a Precellys evolution tissue lyser homogenizer. The protease activity (%) in plasma was established as described by Ramos-Pinto et al. [54] and calculated in comparison to the reference sample. The anti-protease activity (%) in plasma was determined according to Machado et al. [52]; however, the incubation with the phosphate buffer (NaH_2_PO_4_, Sigma) and azocasein (Azocasein, Sigma) was performed for 1 h, at 22 °C, in the dark. The anti-protease activity inhibition was calculated in comparison to the reference sample. The total peroxidase activity in the plasma and intestine (units mL^−1^) was quantified following the protocol described by Machado et al. [52], and calculated by defining one unit of peroxidase as that which produces an absorbance change of 1 OD. The total nitric oxide concentration (mg L^−1^) in the plasma and intestine was calculated using a colorimetric Nitrite/Nitrate Assay Kit (Roche Diagnostics GmbH, Mannheim, Germany), adapted for 96-well microplates, as described by Machado et al. [52], and calculated based on the standard curve. Immunoglobulin M levels (Absorbance OD_450nm_) in the plasma and intestine were analyzed by an enzyme-linked immunosorbent assay (ELISA) according to Cuesta et al. [55] and adapted by Ramos-Pinto et al. [56]; however, we diluted 5 µL of plasma in 495 µL of sodium carbonate buffer (Na_2_CO_3_, 50 mM, pH = 9.6, Sigma). In the case of the intestine, 35 µL of homogenates was dissolved (1:10) in 315 µL of sodium carbonate (Na_2_CO_3_, 50 mM, pH = 9.6, Sigma), and the primary antibody was dissolved in a 1:200 proportion. All the analyses were conducted in triplicates. 

### 2.6. Liver and Intestinal Oxidative Stress

Liver and gut tissues were homogenized (1:10) with K phosphate buffer (KPB) (K_2_HPO_4_ 0.1 M, KH_2_PO_4_ 0.1 M, pH 7.4, Sigma) using a Precellys evolution tissue lyser homogenizer. Aliquots of 200 µL from homogenates were used to determine lipid peroxidation, and 4 µL of 4% 3,5-Di-tert-4-butylhydroxytoluene (BHT, in methanol, Sigma) was added to each sample to prevent artefactual lipid oxidation, as described by Torres et al. [57]. The remaining homogenates were centrifuged at 10,000× *g* for 20 min and 4 °C, and the supernatant was stored at −80 °C until assayed.

The lipid peroxidation (nmol g^−1^) was determined with a microplate reader as adapted by Almeida et al. [58] by measuring thiobarbituric acid-reactive substances (TBARS) as suggested by Bird and Draper [59], with some adaptations performed by Peixoto et al. [60]. Briefly, the homogenates were incubated with 100 μL of TCA 100% and 1 mL of TBA 0.73%, Tris-HCL, and DTPA (Sigma and Fluka) solution at 100 °C for 60 min. The samples were then centrifuged at 11,500× *g* for 5 min and 200 μL of the supernatant was added to the microplate wells. Absorbance was measured at 535 nm.

The colorimetric Pierce^TM^ BCA Protein Assay Kit (Thermo Scientific^TM^, Waltham, MA, USA) was used to determine the total protein concentration (mg mL^−1^), as described by Peixoto et al. [60]. In total, 10 μL of each sample was diluted (1:50) in 490 μL of K-phosphate buffer (0.1 M; pH 7.4) and 25 μL of each diluted sample was plated in microplate wells. Then, 200 μL of the reaction buffer was added to each well and the absorbance was read at 562 nm in a Synergy HT microplate reader. Bovine serum albumin (BSA, 2 mg mL^−1^) was used as standard.

The catalase activity (U mg^−1^ of protein) was determined by measuring the consumption of hydrogen peroxide (H_2_O_2_), as described by Clairborne [61], adapted to the microplate reader by Almeida et al. [58] and adjusted by Peixoto et al. [60]. Briefly, the samples were diluted to 0.7 mg mL^−1^ of protein in K-phosphate buffer (0.1 M; pH 7.4) and 10 μL of each diluted sample was plated in a UV microplate. After, 150 μL of the reaction buffer (K-phosphate buffer (0.05 M pH 7.0) and H_2_O_2_ 30%) was added to each well and the absorbance was read at 240 nm for 2 min (1 read every 15 s) in a Synergy HT microplate reader.

The total glutathione levels (nmol mg^−1^ of protein) were determined according to Griffith [62], adapted to the microplate reader by Baker et al. [63], with some modifications suggested by Peixoto et al. [60]. The samples were diluted to 0.7 mg mL^−1^ of protein in K-phosphate buffer (0.1 M; pH 7.4) and 50 μL of each diluted sample was plated to microplate wells. After, 250 μL of the reaction buffer (K-phosphate buffer (0.2 M, pH 8.0), NADPH (β-nicotanimide adenine dinucleotide 2′-phosphate reduced tetrasodium salt; Alpha Aesar, Tewksbury, MA, USA), DTNB, and glutathione reductase (Sigma)) was added to each well and the absorbance was read at 412 nm for 3 min (1 read every 20 s) in a Synergy HT microplate reader.

The glutathione S-transferase activity (mU mg^−1^ of protein) was quantified by the conjugation of reduced glutathione with 1-chloro-2,4-dinitrobenzene (CDNB) according to Habig et al. [64] and adapted to a microplate reader by Frasco and Guilhermino [65], with the following adaptions by Peixoto et al. [60]. The samples were diluted to 0.7 mg mL^−1^ of protein in K-phosphate buffer (0.1 M; pH 7.4) and 50 μL of each diluted sample was plated to microplate wells. After, 250 μL of the reaction buffer (K-phosphate buffer (0.2 M, pH 6.5), GSH, and CDNB solutions) was added to each well and the absorbance was read at 340 nm for 5 min (1 read every 20 s) in a Synergy HT microplate reader.

Superoxide dismutase activity (U mg^−1^ of protein) was measured according to Flohe and Otting [66], adapted to the microplate reader by Lima et al. [67], with some modifications. Briefly, the samples were adjusted to a 0.3 mg mL^−1^ protein concentration, with KPB and 50 µL of these suspensions were added to flat-bottomed 96-well plates. KPB was used instead of liver PMS as a blank. Then, 200 µL of the reaction solution (0.064 mmol L^−1^ of xanthine in 1 mmol L^−1^ of NaOH and 0.0273 mmol L^−1^ cytochrome c in 50 mmol L^−1^ Na-phosphate buffer (pH 7,8) with 1 mmol L^−1^ Na-EDTA), and 50 µL of xanthine oxidase solution (0.03 U mL^−1^) were added to each well. The final assay concentrations per well were 0.042 mmol L^−1^ of xanthine, 0.018 mmol L^−1^ cytochrome c, 0.005 U mL^−1^ xanthine oxidase solution, 0.06 mmol L^−1^ of NaOH, 30 mmol L^−1^ of NaH_2_PO_4_ and Na_2_HPO_4_, and 0.62 mmol L^−1^ Na-EDTA. The reaction was monitored by the formation of superoxide anion at 550 nm every 20 s for 3 min on a Synergy^TM^ HT microplate reader (Bio-Tek^®^, Winooski, VT, USA). Enzyme activity is expressed as enzyme units per milliliter of total protein (U mL^−1^ protein). All analyses were conducted in triplicates. 

### 2.7. Intestinal Gene Expression

The mRNA expression of the anterior portions of the intestine were analyzed to evaluate the innate immunity, oxidative stress, inflammation, and DNA damage. For total RNA isolation, the intestines were placed in a 2 mL tube containing 0.5 mL of Trizol (Nzol Reagent, NZYTech, Lisbon, Portugal) and 2 zirconium oxide-coated ceramic grinding sphere (2 mm diameter) and homogenized in a Precellys 24 tissues homogenizer (Bertin Ins., Montigny-le-Bretonneux, France) by 2 cycles of 6000× *g* for 20 s. After, 150 µL of chloroform was added to the homogenate, gently vortexed, and centrifuged for 15 min at 12,000× *g* and 4 °C. Around 300 µL of the aqueous phase was transferred to a new 1.5 mL tube with 300 µL of 70% ethanol and gently homogenized. After this step, the RNA extraction was carried out with a NZY Total RNA Isolation Kit (NZYTech, Lisbon, Portugal) following the manufacturer’s specifications. The quantification and purity of RNA were assessed by spectrophotometry and the 260:280 and 260:230 ratios were determined. RNA integrity was verified through 1.5% agarose gel electrophoresis. First-strand cDNA was synthesized with a NZY First-Strand cDNA Synthesis Kit (NZYTech, Lisbon, Portugal) following the manufacturer’s specifications. Primers were selected using Genebank and other publications and are described in Table 2. Efficiencies were calculated in serial five-fold dilutions of cDNA, using the slope of the regression line of the cycle thresholds (Ct) versus the relative concentration of cDNA as described by Machado et al. (2018) [68]. The amplification of primer dimers was also verified through melting curve analysis. Quantitative PCR assays were performed with an iQ5 Real Time PCR detection System (Bio-Rad, Hercules, CA, USA) using 4.4 µL of diluted cDNA (1:50 dilution) mixed with 5 µL of iQ SYBR green 2× Supermix (Bio-Rad, Hercules, CA, USA) and 0.3 µL (10 mM) of each specific primer in a final volume of 10 µL. The standard cycling conditions were initially, one cycle of 95 °C for 10 min, one cycle of 95 °C for 15 s and one cycle of MT for 1 min, followed by 40 cycles of 95 °C for 15 s, one cycle of 95 °C for 1 min, and one cycle of the MT for 30 s, followed by a melting curve from MT to 95 °C, with increments of 0.5 °C for each 0.5 s, and finally a cycle of 95 °C for 15 s. All reactions were carried out as technical duplicates. The expression of the target gene was normalized using the expression of the 18S gene.

### 2.8. Statistical Analysis

All the data were expressed as means (M) and standard deviation (SD) and analyzed for normality and homogeneity of variance. When required, results were log transformed before being treated statistically to ensure normality and homogeneity of the data. Outliers were removed from the analysis. For each trial, one-way ANOVA was used, and the Tukey post hoc test was used to identify differences in the experimental treatments. All the statistical analysis was performed using IBM SPSS Statistics 24. The level of significance for all statistical tests used was *p* < 0.05.

## 3. Results

### 3.1. Growth Performance

**Figure 2:**
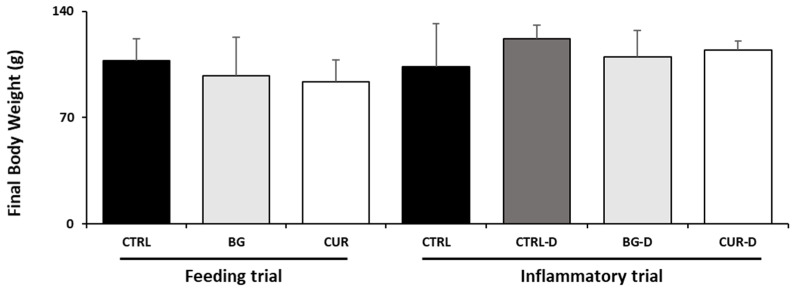
Final body weight of gilthead seabream after the feeding and inflammatory trials (*n* = 12). CTRL (control diet), BG (β-glucans diet), CUR (curcumin diet), CTRL-D (control diet + dextran sodium sulphate − DSS), BG-D (β-glucans diet + DSS), CUR-D (curcumin diet + DSS).

Growth performance was evaluated during the feeding and inflammatory trials. The feeding behavior of the juvenile gilthead seabream did not change during the trials, and the entire feed rations were consumed. No statistical differences were found in the final body weight between the dietary treatments of both trials (Figure 2). 

### 3.2. Hematological Profile

The effects of different dietary treatments on the hematological profile are described in Table 3. While no significant differences were found among dietary treatments at the end of the feeding trial, DSS increased RBC and the peripheral thrombocyte counts in fish fed CTRL-D. In contrast, the HG concentration dropped in fish fed CTRL-D compared to their counterparts fed CUR-D. A synergistic effect of DSS and dietary additives was observed for HT with increased values in fish fed BG-D and CUR-D compared to non-stimulated animals (i.e., CTRL). A similar effect was observed for peripheral lymphocytes in seabream fed BG-D compared to unstimulated fish fed CTRL. Moreover, MCV decreased in seabream fed CTRL-D compared to CTRL while no changes were observed in those fish fed BG-D and CUR-D dietary treatments. Fish fed BG-D also augmented MCH compared to those fish fed CTRL-D.

### 3.3. Plasma Humoral Immunological Parameters

**Figure 3:**
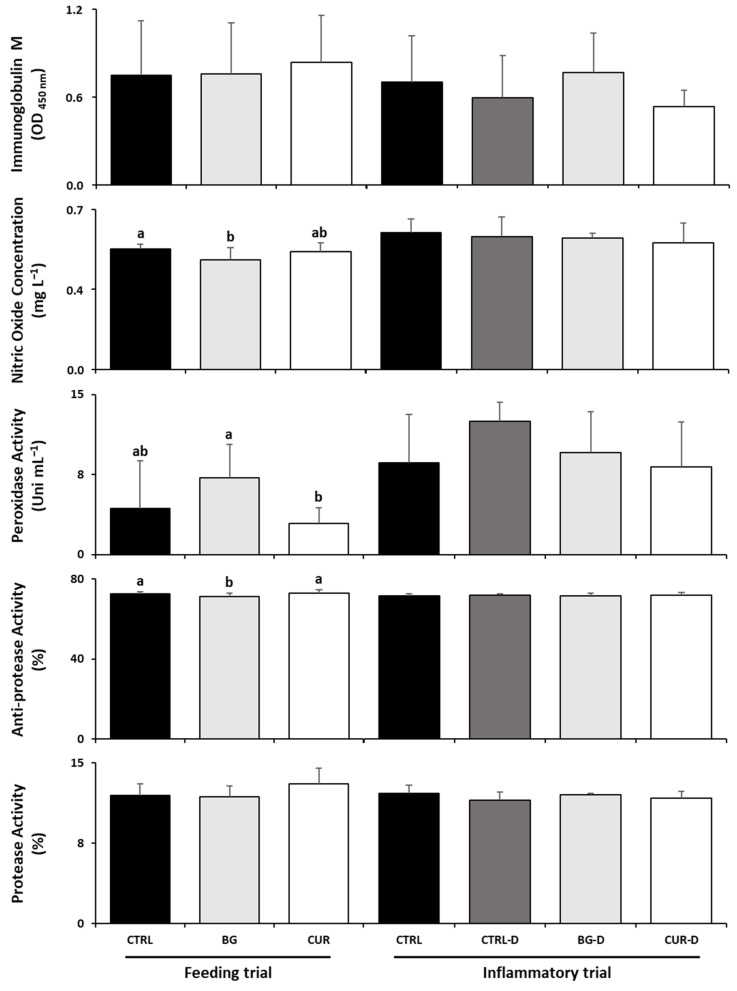
Humoral immunological parameters in plasma of gilthead seabream after the feeding and inflammatory trials (*n* = 12). CTRL (control diet), BG (β-glucans diet), CUR (curcumin diet), CTRL-D (control diet + dextran sodium sulphate − DSS), BG-D (β-glucans diet + DSS), CUR-D (curcumin diet + DSS). Different letters mean significant differences among dietary treatments (*p* < 0.05).

After the feeding trial, seabream fed BG had decreased plasma anti-protease activity and nitric oxide levels compared to fish fed the CTRL diet while fish fed CUR showed lower plasma peroxidase levels than seabream fed BG. After the gut inflammation trial, no significant differences were observed among the dietary treatments in the plasma humoral immunological parameters (Figure 3).

### 3.4. Liver Oxidative Stress

**Figure 4:**
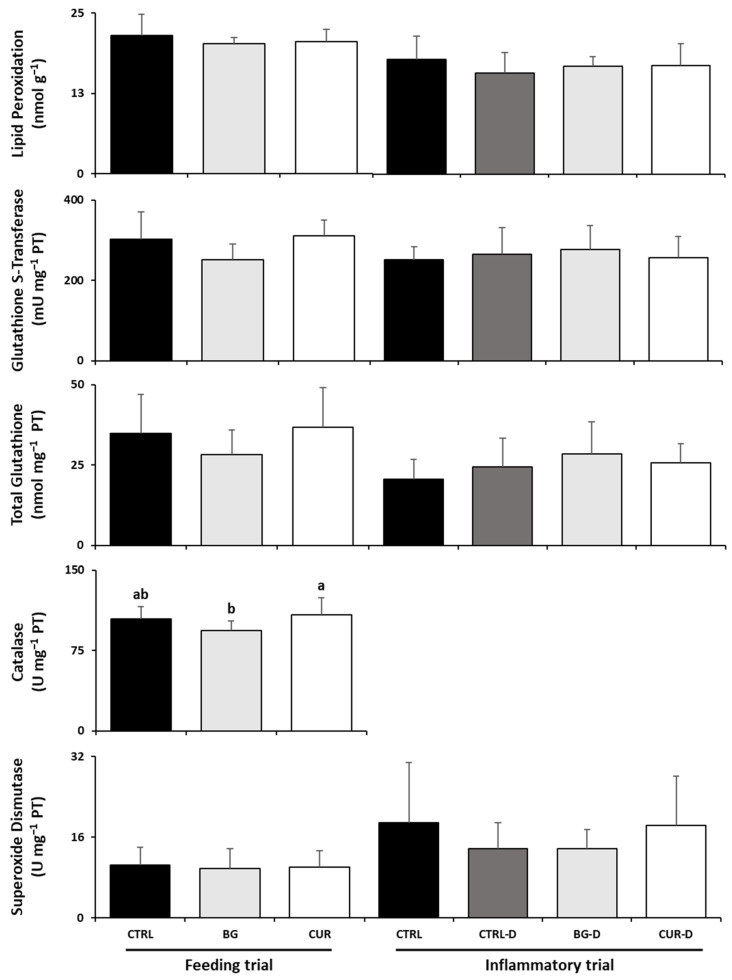
Liver oxidative stress biomarkers of gilthead seabream after the feeding and inflammatory trials (*n* = 12). CTRL (control diet), BG (β-glucans diet), CUR (curcumin diet), CTRL-D (control diet + dextran sodium sulphate − DSS), BG-D (β-glucans diet + DSS), CUR-D (curcumin diet + DSS). Different letters mean significant differences among dietary treatments (*p* < 0.05).

As Figure 4 shows, there were no significant differences among fish fed the experimental diets and their counterparts fed the CTRL diet at the end of the feeding trial. However, fish fed CUR had significantly increased hepatic catalase activity compared to seabream fed BG. Likewise, after the gut inflammation trial, there were no significant differences between the dietary treatments in the liver oxidative stress parameters.

### 3.5. Intestinal Humoral Immunological Parameters

**Figure 5:**
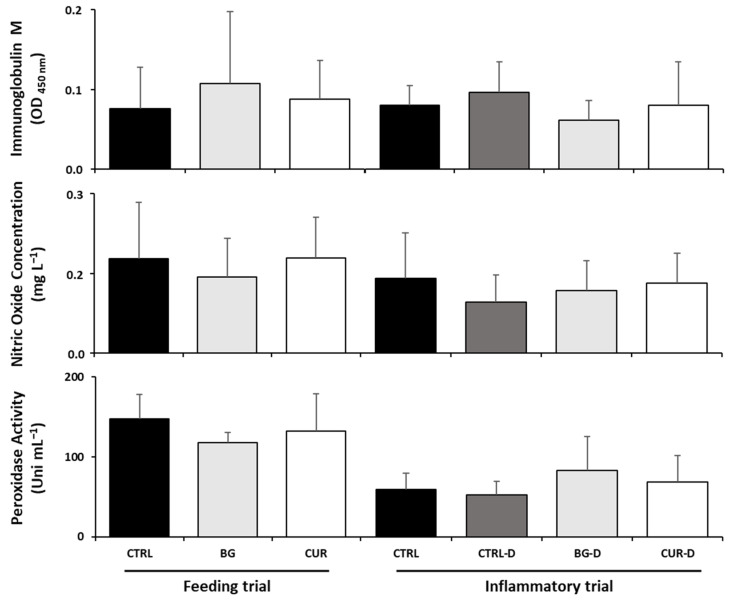
Intestinal humoral immunological parameters of gilthead seabream after the feeding and inflammatory trials (*n* = 12). CTRL (control diet), BG (β-glucans diet), CUR (curcumin diet), CTRL-D (control diet + dextran sodium sulphate − DSS), BG-D (β-glucans diet + DSS), CUR-D (curcumin diet + DSS).

No significant differences were found in the intestinal humoral immunological parameters among seabream fed dietary treatments at the end of the feeding trial (Figure 5), although the gut peroxidase activity and oxide nitric levels tended to drop in the fish fed the BG diet. Similarly, no changes were observed among dietary treatments after the gut inflammation trial.

### 3.6. Intestinal Oxidative Stress

**Figure 6:**
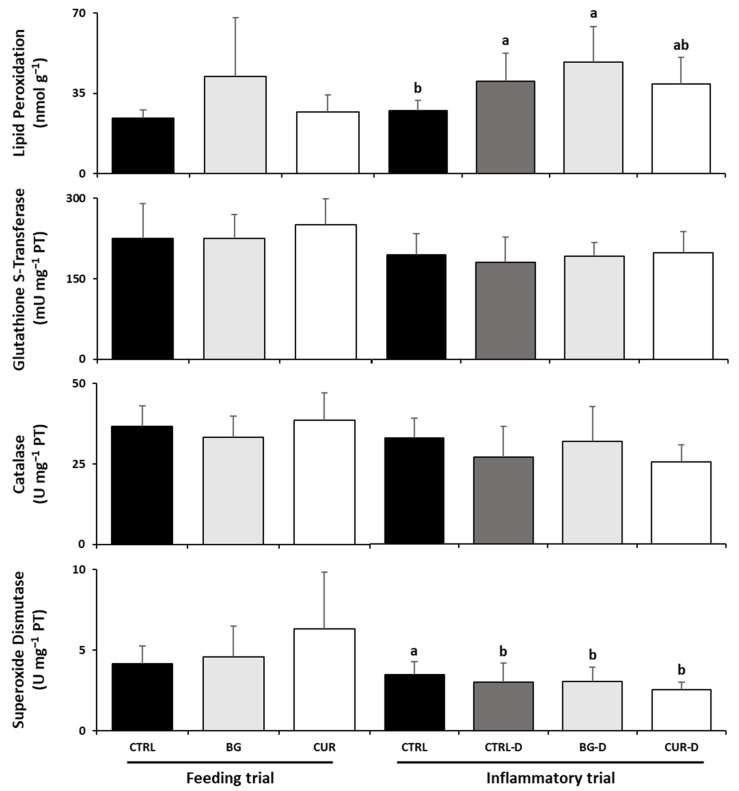
Intestinal oxidative stress biomarkers of gilthead seabream after the feeding and inflammatory trials (*n* = 12). CTRL (control diet), BG (β-glucans diet), CUR (curcumin diet), CTRL-D (control diet + dextran sodium sulphate − DSS), BG-D (β-glucans diet + DSS), CUR-D (curcumin diet + DSS). Different letters mean significant differences among dietary treatments (*p* < 0.05).

Gilthead seabreams were not affected by dietary treatments at the end of the feeding trial (Figure 6). However, the gut inflammation induced by DSS affected the intestinal oxidative stress biomarkers by decreasing the superoxide dismutase activity in fish fed CTRL-D, BG-D, and CUR-D compared to those fed CTRL. While lipid peroxidation levels increased in the gut of seabream fed either CTRL-D or BG-D compared to CTRL, those levels dropped in fish fed CUR-D, although this was not significantly different compared to their counterparts fed CTRL-D and BG-D.

### 3.7. Intestinal Gene Expression

**Figure 7:**
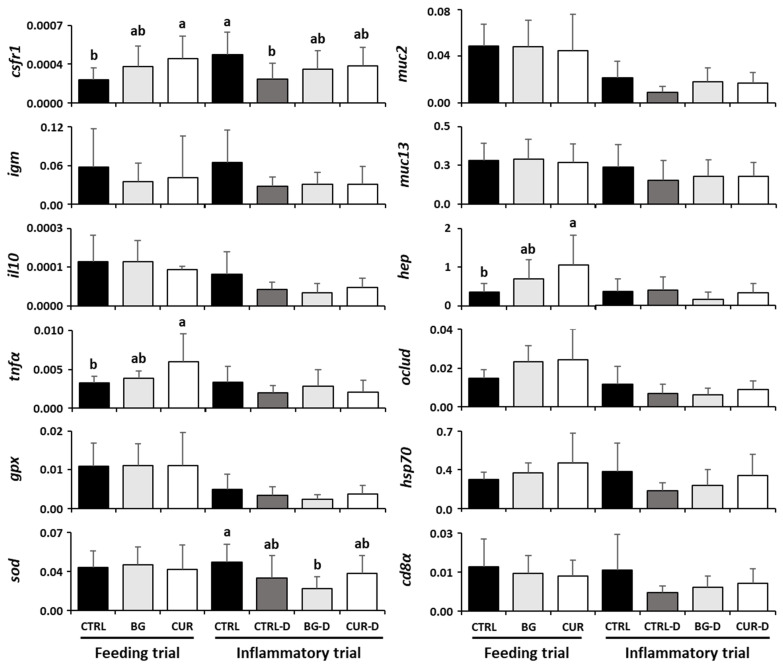
Intestinal gene expression profiles of gilthead seabream after the feeding and inflammatory trials (*n* = 12). CTRL (control diet), BG (β-glucans diet), CUR (curcumin diet), CTRL-D (control diet + dextran sodium sulphate − DSS), BG-D (β-glucans diet + DSS), CUR-D (curcumin diet + DSS). Different letters mean significant differences among dietary treatments (*p* < 0.05).

The gene expression profiles in the intestine of gilthead seabream after both the feeding and the gut inflammation trials are described in Figure 7 and Supplementary Table S1. Fish fed the CUR dietary treatment presented an upregulation of the *tnfα*, *csf1r*, and *hep* genes compared to their counterparts fed the CTRL diet. Following the gut inflammation trial, most target genes showed a tendency to be downregulated by the DSS treatment. However, only *csf1r* transcripts were significantly decreased in fish fed CTRL-D compared to specimens fed CTRL. Moreover, *sod* mRNA expression was also significantly decreased in seabream fed the BG-D dietary treatment compared to fish fed CTRL (Figure 7).

## 4. Discussion

Preventing fish diseases and economic losses is crucial in order to increase the profitability and sustainability of the aquaculture industry [1,2,3]. The use of functional feeds can improve fish health and disease resistance, acting as prophylactic alternatives to antibiotics and others chemotherapeutics [4,30,33]. Some feed additives, such as β-glucans and curcumin, have multiple beneficial effects on fish, potentiating growth, physiological functions, immunity, antioxidant activity, and disease resistance [2,4,40,45,50,51]. In the present study, while some minor changes were observed at the systemic level in fish fed dietary treatments at the end of the feeding trial, health-promoting effects were evident at the local level (i.e., gut) due to dietary curcumin supplementation. The increase in intestinal *tnfα*, *csf1r*, and *hep* in fish fed CUR suggests activation of the innate immune response and mobilization of intraepithelial macrophages. Although the response of fish to the dietary treatment with feed additives may not be ample in the absence of a stressor [34], curcumin is known to have immunomodulatory properties in fish [41,42,43,45,46,47].

Another biological property associated with curcumin and β-glucans is their anti-inflammatory capacity [51,69]. Therefore, this study also aimed to evaluate the ability of these two feed additives to minimize DSS inflammatory effects since DSS is a common method used to study the intestinal inflammatory process in several animal models [11,12,13,14,15,16,17,18], including in fish [19,20,21,22,23,24,25,26], and feed additives can be used to reverse the effects of some intestinal inflammatory compounds [70].

Intestinal inflammation is a defensive process to control microbial infections and tissue damage [71]. However, when dysregulated, intestinal inflammation involves a dysfunctional response of the host towards the diet, bacteria, and chemicals [22,23,25,71], with the overproduction of pro-inflammatory cytokines, such as TNFα, IL1β, and IFNγ, triggered by the activation of the NFκB pathway, which causes the collapse of the intestinal barrier [71,72]. According to previous studies in other animal models, intestinal inflammation induced by DSS is related to the overexpression of *tnfα*, *il-1β*, *il-6*, and *il-17* [11,14,15,16,18,19,21]. Nonetheless, in this study, the expression of *tnfα* did not differ significantly among dietary treatments. The effectiveness of the DSS-induced inflammation depends on the concentration, duration, frequency, and molecular weight of the DSS administration but also the species and susceptibility of the animal model [73]. In the present study, the dosage of DSS used did not appear to produce significant extensive inflammation in the intestine of gilthead seabream. According to Oehlers et al. [19] and Oehlers et al. [20], an immersion with 0.5% (*w*/*v*) DSS is sufficient to induce intestinal inflammation in zebrafish larvae, whereas an oral perfusion with 5% DSS [21] can also be used in pufferfish.

In this study, a clear tendency to decrease intestinal *hsp70*, *muc2*, and *igm* transcripts may suggest that DSS can also induce harmful effects in the gut of seabream to some extent, a fact further corroborated by the significant increase in gut lipid peroxidation. The gastrointestinal tract has a protective mucus barrier, where mucins protect the surface of the intestinal epithelial cells [24,73]. Mucin depletion, epithelial degeneration, necrosis, and inflammation are the major histologic features found in the intestine after the ingestion of DSS in mice and pigs [27,74]. Heat shock proteins (HSPs) are involved in the regulation of the intestinal immune functions [24], maintaining gastrointestinal homeostasis [75], and protecting the tissues from stressors [76]. When HSP levels are reduced, the severity of the intestinal inflammation is enhanced [75]. In the present study, BG-D and CUR-D seemed to partially restore the drop in *muc2* and *hsp70* mRNA expression due DSS treatment, an interesting fact that should be further explored in future trials since there is potential in the use of these dietary treatments in seabream. Carballo et al. [37] also verified an improved expression of *hsp90* in the kidney and spleen of sole after intraperitoneal injections with chrysolaminarin. Increased expression of the *hsp70* gene and HSP70 protein was also found in rat neuronal cells treated with curcumin [77].

Curcumin is a powerful anti-inflammatory compound that modulates many inflammatory mediators, decreasing the response of the NF-kB signaling pathway and, consequently, reducing the production of inflammatory cytokines (TNFα, IL1β, IL2, IL6, IL8, and IL12) [40,71,72,78,79,80]. IL10 is an anti-inflammatory cytokine and a major regulator of intestinal homeostasis [17,24]. DSS is associated with the downregulation of intestinal *il10* in mice Silvestri [16], as we found in this study, though with no statistical differences to CTRL. However, curcumin was able to improve the expression of *il10*, though without statistical differences to CTRL-D.

Inflammation and oxidative stress are pathological processes known to be related [72]. The inflammatory cells release a high number of oxygen and nitrogen reactive species (ROS and NOS) in the inflammation site, leading to oxidative stress [72]. On the other hand, oxidative stress triggers an intracellular signaling cascade that enhances the pro-inflammatory gene expression [72]. The release of ROS and NOS, and further intestinal inflammation, results in intestinal ischemia and necrosis, creating a vicious cycle [71]. As a result, inflammation-related signaling pathways are upregulated, such as the NFkB pathway [78]. At low concentrations, ROS are an indispensable defense mechanism to control microorganisms; however, inadequate removal of their intermediate compounds can lead to oxidative stress [81]. The antioxidant nonenzymatic (total glutathione) and enzymatic (superoxide dismutase, catalase, glutathione peroxidase, glutathione s-transferase, and glutathione reductase) systems are the first lines of defense against oxidative stress [81]. Superoxide dismutase and glutathione peroxidase activities are crucial to stabilize cell membranes and to protect the intestinal tissue from the homeostasis instability caused by excessive production of ROS and NOS, but superoxide dismutase and glutathione peroxidase activities decreased in the intestine after the ingestion of DSS in mice [18]. Without this antioxidant capacity, the intestine is not able to repair the intestinal wall and alleviate the inflammation [18].

In the present study, superoxide dismutase activity and the *sod* and *gpx* mRNA levels decreased in the intestine of seabream orally administered DSS, thus hampering the antioxidant capacity of the intestine to overcome the production of associated ROS and NOS, allowing a significant accumulation of free radicals in the intestine, and contributing to the lipid peroxidation [78]. In fact, lipid peroxidation was significantly increased in fish fed CTRL-D and BG-D while curcumin was able to improve the intestinal response to lipid peroxidation induced by the DSS chemical and tended to increase the *sod* and *gpx* transcripts. Curcumin has a strong ability to stimulate antioxidant enzyme activities and to inhibit lipid peroxidation [40,44,45,47,50,51,78,79,80,81,82,83,84,85], reducing the levels of ROS and NOS, responsible for the breakdown of intestinal tight junctions, tissue injury, and necrosis [71]. An increase in the relative mRNA expression of *sod*, *cat*, *gpx*, *gr*, and *gst* after curcumin supplementation was found in previous studies [44,78]. The antioxidant mechanisms of curcumin in the gene expression seem to be related to the activation of the nuclear factor erythroid 2-related factor (NRF2) signaling pathway, which plays a major role in improving the gene expression of codifying genes for detoxifying, antioxidant, and anti-inflammatory proteins [44,51,76].

Intestinal inflammation, necrosis, and oxidative stress impair the intestinal barrier and the microbiota community (dysbiosis), allowing bacterial translocation and, ultimately, systemic inflammation [71,73]. The hematological parameters are an important tool to establish fish physiological status and health and provide important information for the diagnosis and prognosis of diseases [86]. Hemograms are related to erythropoiesis and anemia [86] while leucograms are indispensable for evaluating stress and diseases in fish [86,87]. The presence of high numbers of circulating leukocytes is indicative of inflammation or infection [86,87]. In the present study, hematocrit and red blood cell counts were increased after the dietary treatment with DSS while hemoglobin was reduced. β-glucans and curcumin improved the hematocrit and hemoglobin content, indicating that these feed additives had a beneficial effect on the metabolism and availability of nutrients in the bloodstream [3], contributing with more oxygen and nutrients to the cells at the inflammation site [86,87]. An enhancement of the hematological parameters can be found after dietary supplementation with curcumin in fish [3,43,45,47,50,84]. An improvement in hemoglobin production was also found in broilers by Sugiharto et al. [88] after the ingestion of curcumin, though the concentration of erythrocytes and hematocrit were not affected by this feed additive. On the other hand, laminarin was able to reduce the red blood cell counts and hemoglobin content in fish infected with *Aeromonas hydrophila* [89]. As DSS inflammation impairs the intestinal barrier and may allow bacterial translocation, an increase in the white blood cell counts in plasma is essential to control the bacterial population and prevent systemic infection [3]. In this study, the increase in circulating thrombocytes in fish fed CTRL-D and BG-D suggests a certain degree of thrombocytes proliferation and mobilization due to the DSS treatment, a fact that seems to be counteracted by dietary curcumin. Moreover, the BG-D dietary treatment was able to increase circulating lymphocytes and further suggests certain immunomodulatory properties of BG in response to the DSS treatment that deserve further attention.

As described by Nordvi et al. [70], there is a lack of studies related to intestinal damage and inflammation in fish models, especially low and moderate reversible inflammation. In this study, DSS produced mild effects on gilthead seabream health parameters, and, therefore, it can be suggested as a good model to study the intestinal inflammatory process in fish. However, other studies should be conducted to establish DSS’ dose and effect to define DSS as a robust model to study fish intestinal inflammation. Additionally, this study showed that the conjugation of these two feed additives seems to be promising, reverting mild signs of inflammation and strengthening the defense mechanisms of gilthead seabream towards pathogens, improving the response to intestinal inflammation, and preventing the transition of bacteria from the intestine into the bloodstream; however, further studies are needed to establish the concentration and fully understand the mechanisms involved in this process.

## 5. Conclusions

In conclusion, dietary administration of DSS affected the health parameters of gilthead seabream, such as the hematological profile, intestinal oxidative stress biomarkers, and intestinal gene expression profile, indicating that DSS is a useful model for studying intestinal inflammation in fish. Additionally, the adverse effects induced by DSS seemed to be partially alleviated by dietary supplementation with curcumin, which improved the hematological profile and helped against oxidative stress. However, further studies are needed to fully understand the correct amount of DSS as a chemical inducer of intestinal inflammation, the feed additive concentration necessary to prevent intestinal inflammation, and the associated mechanisms involved in this process.

## Figures and Tables

**Table 1:** Ingredient and chemical composition of the diets (feeding trial).

Ingredients (% Feed) *	Control Diet	β-Glucans Diet	Curcumin Diet
Fishmeal super prime	13.00	13.00	13.00
Fishmeal 60	7.00	7.00	7.00
CPSP 90	2.00	2.00	2.00
Poultry meal 65	15.00	15.00	15.00
Soy protein concentrate	5.00	5.00	5.00
Wheat gluten	4.20	4.20	4.20
Corn gluten meal	8.00	8.00	8.00
Soybean meal 48	10.00	10.00	10.00
Rapeseed meal	5.00	5.00	5.00
Sunflower meal	5.00	5.00	5.00
Wheat meal	11.13	10.13	10.13
Vitamin and mineral premix	1.00	1.00	1.00
Vitamin C35	0.05	0.05	0.05
Vitamin E50	0.02	0.02	0.02
Betaine HCl	0.20	0.20	0.20
Antioxidant	0.20	0.20	0.20
Fish oil	3.96	3.96	3.96
Soybean oil	5.28	5.28	5.28
Rapeseed oil	3.96	3.96	3.96
Curcumin extract 95%			0.20
Algae beta-glucans extract		1.00	
Proximate Analyses (% dry matter)			
Moisture	7.07		
Crude protein	46.16		
Crude fat	17.80		
Ash	8.34		
Gross energy (MJ Kg^−1^)	21.22		

* Fishmeal super prime: 66.3% CP, 11.5% CF, Pesquera Diamante, Peru; Fishmeal 60: CONRESA 60–65% CP, 10% CF, Conserveros Reunidos S.A., Spain; CPSP 90: 86% CP, 6% CF, Sopropêche, France; Poultry meal 65: 65% CP, 12% CF, SAVINOR UTS, Portugal; Soy protein concentrate: Soycomil P—62% CP, 0.5% CF, ADM Portugal, Portugal; Wheat gluten: VITEN—81% CP, 2.1% CF, Roquette, France; Corn gluten meal: 58% CP, 4% CF, MPS, France; Soybean meal 48: solvent extracted soybean meal—43% CP, 2.7% CF, CARGILL, Spain; Rapeseed meal: defatted rapeseed meal—32.7% CP, 4.1% CF, Ribeiro & Sousa Lda, Portugal; Sunflower meal 40: solvent extracted dehulled sunflower meal—43% CP, 3% CF, MAZZOLENI SPA, Italy; Wheat meal: 10.2% CP; 1.2% CF, MOLISUR, Spain; Vitamin and mineral premix: PREMIX Lda, Portugal—Vitamins (IU or mg kg^−1^ diet): DL-alpha tocopherol acetate, 100 mg; sodium menadione bisulphate, 25 mg; retinyl acetate, 20,000 IU; DL-cholecalciferol, 2000 IU; thiamin, 30 mg; riboflavin, 30 mg; pyridoxine, 20 mg; cyanocobalamin, 0.1 mg; nicotinic acid, 200 mg; folic acid, 15 mg; ascorbic acid, 500 mg; inositol, 500 mg; biotin, 3 mg; calcium pantothenate, 100 mg; choline chloride, 1000 mg, betaine, 500 mg. Minerals (g or mg kg^−1^ diet): copper sulphate, 9 mg; ferric sulphate, 6 mg; potassium iodide, 0.5 mg; manganese oxide, 9.6 mg; sodium selenite, 0.01 mg; zinc sulfate, 7.5 mg; sodium chloride, 400 mg; excipient wheat middling’s; Vitamin C35: ROVIMIX STAY-C 35, DSM Nutritional Products, Switzerland; Vitamin E50: ROVIMIX E50, DSM Nutritional Products, Switzerland; Betaine HCl: ORFFA, The Netherlands; Antioxidant: VERDILOX PX, KEMIN EUROPE NV, Belgium; Fish oil: Sopropêche, France; Soybean oil: J.C. Coimbra, Portugal; Rapeseed oil: J.C. Coimbra, Portugal; Curcumin extract 95%: Denk Ingredients (Munich, Germany); Algae beta-glucans extract: beta-glucans extract from microalgae (*P. tricornutum*).

**Table 2:** Oligonucleotide primers information used for real-time PCR analysis of gene expression.

Target Gene Name	Symbol	Accession nr.	Forward Primer (5′-3′) Reverse Primer (5′-3′)	PL ^1^	MT ^2^	E ^3^
Mn superoxide dismutase	*sod*	JQ308833	CCTGACCTGACCTACGACTATGG AGTGCCTCCTGATATTTCTCCTCTG	134	60	2.05
Glutathione peroxidase 1	*gpx*	DQ524992	GAAGGTGGATGTGAATGGAAAAGATG CTGACGGGACTCCAAATGATGG	129	60	2.21
Tumor necrosis factor alpha	*tnfα*	AJ413189.2	TGAACAGAGGCGACAAACTG GCCACAAGCGTTATCTCCAT	245	60	1.92
Interleukin-10-like	*il10*	XM_30418889.1	AACATCCTGGGCTTCTATCTG GTGTCCTCCGTCTCATCTG	65	57	2.07
Immunoglobulin M heavy chain	*igm*	AM493677	CAGCCTCGAGAAGTGGAAAC GAGGTTGACCAGGTTGGTGT	136	59	2.19
Macrophage colony stimulating factor receptor	*csf1r*	AM050293	ACGTCTGGTCCTATGGCATC AGTCTGGTTGGGACATCTGG	129	60	2.11
CD8 alpha chain precursor	*cd8α*	AJ878605	CTCGACTGGTCGGAGTTAA TCCATCAGCGGCTGCTCGT	287	60	1.91
Heat shock protein 70	*hsp70*	DQ524995.1	ACGGCATCTTTGAGGTGAAG TGGCTGATGTCCTTCTTGTG	124	55	2.05
Occludin	*ocln*	KF861990.1	TCATCTCCTACCCTCCAGTCA ATGGTCTGCTTGTGGTCCTC	96	60	2.03
Hepcidin 1	*hep*	EF625901	GCCATCGTGCTCACCTTTAT CCTGCTGCCATACCCCATCTT	382	60	2.07
Mucin-13-like	*muc13*	XM030399162	TTCAAACCCGTGTGGTCCAG GCACAAGCAGACATAGTTCGGATAT	67	60	1.96
Mucin-2-like	*muc2*	XM_030425504.1	GTGTGTGGCTGTGTTCCTTGCTTTGT GCGAACCAGTCTGGCTTGGACATCA	67	60	1.98

^1^ Product length (Amplicon (nt)), ^2^ Melting temperature, ^3^ Efficiency of PCR reactions.

**Table 3:** Hematological profile of gilthead seabream after the feeding and inflammatory trials (*n* = 12). CTRL (control diet), BG (β-glucans diet), CUR (curcumin diet), CTRL-D (control diet + dextran sodium sulphate − DSS), BG-D (β-glucans diet + DSS), CUR-D (curcumin diet + DSS). Values are presented as means ± standard deviation. Different letters mean significant differences among dietary treatments (*p* < 0.05).

Parameters *	Feeding Trial			Inflammatory Trial			
	CTRL	BG	CUR	CTRL	CTRL-D	BG-D	CUR-D
HT (%)	37.4 ± 6.7	36.4 ± 5.7	37.2 ± 6.9	37.0 ^b^ ± 6.4	39.7 ^ab^ ± 3.8	45.3 ^a^ ± 3.6	43.7 ^a^ ± 5.2
RBC (×10^6^ mL)	1.4 ± 0.5	1.3 ± 0.4	1.2 ± 0.6	2.0 ^b^ ± 0.9	2.9 ^a^ ± 0.8	2.3 ^ab^ ± 0.7	2.7 ^ab^ ± 0.1
HG (g dL^−1^)	2.4 ± 1.0	2.6 ± 1.2	2.0 ± 0.7	2.0 ^ab^ ± 0.3	1.8 ^b^ ± 0.6	2.3 ^ab^ ± 0.8	2.9 ^a^ ± 1.1
MCV (µm^3^)	273.4 ± 72.8	310.1 ± 100.9	362.7 ± 209.6	209.7 ^a^ ± 70.9	134.7 ^b^ ± 27.1	190.1 ^a^ ± 48.3	162.1 ^ab^ ± 30.8
MCH (pg cell^−1^)	15.0 ± 3.2	16.1 ± 5.2	13.2 ± 3.9	11.5 ^ab^ ± 4.8	7.0 ^b^ ± 2.8	12.8 ^a^ ± 4.3	10.5 ^ab^ ± 3.2
MCHC (g 100 mL^−1^)	6.6 ± 2.7	7.6 ± 4.0	4.8 ± 0.5	5.4 ± 1.4	4.9 ± 1.9	6.3 ± 1.1	6.6 ± 2.0
WBC (×10^4^ mL)	1.9 ± 0.9	2.0 ± 0.7	1.9 ± 0.7	1.7 ± 0.9	2.9 ± 1.7	3.0 ± 1.4	2.1 ± 1.3
Thrombocytes (×10^4^ mL)	0.9 ± 0.4	1.2 ± 0.5	1.0 ± 0.4	0.7 ^b^ ± 0.3	1.6 ^a^ ± 1.0	1.6 ^a^ ± 0.8	1.2 ^ab^ ± 0.7
Lymphocytes (×10^4^ mL)	0.6 ± 0.2	0.6 ± 0.2	0.6 ± 0.2	0.5 ^b^ ± 0.3	0.7 ^ab^ ± 0.3	1.0 ^a^ ± 0.5	0.6 ^ab^ ± 0.4
Monocytes (×10^4^ mL)	0.1 ± 0.1	0.1 ± 0.1	0.1 ± 0.0	0.1 ± 0.1	0.1 ± 0.1	0.1 ± 0.1	0.1 ± 0.0
Neutrophils (×10^4^ mL)	0.1 ± 0.1	0.1 ± 0.1	0.1 ± 0.1	0.1 ± 0.1	0.2 ± 0.1	0.2 ± 0.2	0.1 ± 0.2

* HT (hematocrit), RBC (red blood cells), HG (hemoglobin), MCV (mean corpuscular volume), MCH (mean corpuscular hemoglobin), MCHC (mean corpuscular hemoglobin concentration), WBC (white blood cells).

## Data Availability

The data presented in this study are available on request from the corresponding author.

## Supplementary Materials

The following supporting information can be downloaded at: https://www.mdpi.com/article/10.3390/ani12213019/s1, Table S1: Intestinal gene expression profiles of gilthead seabream after the feeding and inflammatory trials. CTRL (control diet), BG (β-glucans diet), CUR (curcumin diet), CTRL-D (control diet + dextran sodium sulphate − DSS), BG-D (β-glucans diet + DSS), CUR-D (curcumin diet + DSS). Values are presented as means ± standard deviation. Different letters mean significant differences among dietary treatments (*p* < 0.05).
