# Supplementary material for: Dietary Strategies to Modulate the Health Condition and Immune Responses in Gilthead Seabream (Sparus aurata) Juveniles Following Intestinal Inflammation
Source: Animals (Basel). 2022 Nov 3;12(21):3019. doi: 10.3390/ani12213019 (PMC9657010; doi:10.3390/ani12213019)
Supplement: Supplementary file 1 [file animals-12-03019-s001.zip › animals-1982479-supplementary.pdf]

Table S1: Intestinal gene expression profiles of gilthead seabream after the feeding and inflammatory trials. **CTRL** (control diet), **BG** (β-glucans diet), **CUR** (curcumin diet), **CTRL-D** (control diet + dextran sodium sulphate - DSS), **BG-D** (β-glucans diet + DSS), **CUR-D** (curcumin diet + DSS). Values are presented as means ± standard deviation. Different letters mean significant differences among dietary treatments ($p < 0.05$).

Gene expression	Feeding trial			Inflammatory trial			
	CTRL	BG	CUR	CTRL	CTRL-D	BG-D	CUR-D
sod	0.0383 ± 0.0150	0.0412 ± 0.0161	0.0367 ± 0.0218	0.0434^{a} ± 0.0162	0.0294^{ab} ± 0.0200	0.0197^{b} ± 0.0104	0.0333^{ab} ± 0.0161
gpx	0.0108 ± 0.0060	0.0111 ± 0.0056	0.0110 ± 0.0086	0.0049 ± 0.0040	0.0033 ± 0.0023	0.0024 ± 0.0012	0.0037 ± 0.0023
tnfα	0.0033^{b} ± 0.0009	0.0039^{ab} ± 0.0009	0.0060^{a} ± 0.0036	0.0034 ± 0.0020	0.0019 ± 0.0010	0.0028 ± 0.0021	0.0020 ± 0.0016
il10	0.0001 ± 0.0001	0.0001 ± 0.0001	0.0001 ± 0.0000	0.0001 ± 0.0001	0.0001 ± 0.0000	0.0000 ± 0.0000	0.0001 ± 0.0000
igm	0.0581 ± 0.0593	0.0352 ± 0.0290	0.0416 ± 0.0649	0.0537 ± 0.0424	0.0232 ± 0.0116	0.0255 ± 0.0160	0.0261 ± 0.0225
csf1r	0.0002^{b} ± 0.0001	0.0003^{ab} ± 0.0002	0.0004^{a} ± 0.0002	0.0003^{a} ± 0.0001	0.0002^{b} ± 0.0001	0.0002^{ab} ± 0.0001	0.0002^{ab} ± 0.0001
cd8α	0.0141 ± 0.0089	0.0121 ± 0.0056	0.0112 ± 0.0050	0.0131 ± 0.0115	0.0060 ± 0.0020	0.0075 ± 0.0037	0.0088 ± 0.0046
hsp70	0.2583 ± 0.0703	0.3213 ± 0.0886	0.4105 ± 0.2658	0.3349 ± 0.2583	0.1604 ± 0.0711	0.2074 ± 0.1460	0.2976 ± 0.1910
ocln	0.0146 ± 0.0045	0.0233 ± 0.0082	0.0242 ± 0.0157	0.0116 ± 0.0092	0.0068 ± 0.0050	0.0060 ± 0.0036	0.0089 ± 0.0044
hep	0.3523^{b} ± 0.2303	0.6998^{ab} ± 0.4985	1.0486^{a} ± 0.7749	0.3716 ± 0.3151	0.4004 ± 0.3432	0.1642 ± 0.1878	0.3237 ± 0.2378
muc13	0.2820 ± 0.1098	0.2894 ± 0.1285	0.2659 ± 0.1237	0.2385 ± 0.1451	0.1520 ± 0.1272	0.1766 ± 0.1094	0.1776 ± 0.0908
muc2	0.0488 ± 0.0190	0.0482 ± 0.0229	0.0447 ± 0.0312	0.0260 ± 0.0173	0.0108 ± 0.0059	0.0220 ± 0.0138	0.0207 ± 0.0107
